# Laparoscopy-assisted colectomy as an Oncologically safe alternative for patients with stage T4 Colon Cancer: a propensity-matched cohort study

**DOI:** 10.1186/s12885-018-4269-x

**Published:** 2018-04-03

**Authors:** Hao Wang, Xiaoyu Chen, Hao Liu, Tingyu Mou, Haijun Deng, Liying Zhao, Guoxin Li

**Affiliations:** Department of General Surgery, Nanfang Hospital, Southern Medical University, Guangdong Provincial Engineering Technology Research Center of Minimally Invasive Surgery, Guangzhou, 510515 China

**Keywords:** Laparoscopy, Colon cancer, T4 tumor, Propensity score matching, Oncologic outcome, Survival

## Abstract

**Background:**

It is still controversial whether laparoscopy-assisted colectomy (LAC) is suitable for patients with stage T4 colon cancer. This study aimed to compare the short- and long-term outcomes of LAC and open colectomy (OC) for patients with pathologic T4 colon cancer.

**Methods:**

Data of eligible patients with colon cancer in our institution from March 2004 to September 2014 were retrospectively reviewed. The patients were followed up to September 2016. Propensity score matching was performed to control the bias.

**Results:**

Two hundred and forty two patients were selected by propensity score matching, with 121 patients in the LAC group and 121 in the OC group. Mean operating time and rate of intraoperative blood transfusion were similar between two groups. In LAC group, shorter time to first flatus and first liquid intake were observed in patients with pT4b stage disease, but not for patients with pT4a stage disease. Less blood loss and shorter length of postoperative hospital stay were examined in LAC group, including pT4a and pT4b stages. Conversion was required in 9.1% (11 out of 121) cases. DFS and OS were similar between LAC and OC groups. The 5-year DFS rate was 64.2% for pT4a stage and 35.5% for pT4b stage in LAC group, and 62.9% and 33.7% in OC group for pT4a (*p* = 0.374) and pT4b (*p* = 0.385) stage respectively. For 5-year OS rates, two groups did not differ in pT4a stage (LAC 69.2% vs. OC 66.0%, *p* = 0.151) and pT4b stage (LAC 37.5% vs. OC 38.1%, *p* = 0.510).

**Conclusions:**

Laparoscopic colectomy appears to be safe for selected patients with pT4 colon cancer in centres with expertise in minimally invasive surgery.

## Background

Colorectal cancer is the third most common cancer in the worldwide and the fifth leading cause of cancer death with nearly 200,000 deaths in China annually [1, 2]. Surgery remains the major approach to treat colon cancer with curative intent, although chemotherapy probably can decrease disease recurrence and prolong survival time. Laparoscopy-assisted colectomy (LAC), as a minimally invasive approach, is widely used for resectable tumor, mainly including stage T1~T3 tumor, and is attempted for stage T4 tumor including those penetrate serosa (T4a) or invade adjacent structures (T4b) [3–5].

Most of studies comparing LAC and OC for colorectal cancers excluded patients with T4 tumor [5–8]. It is still controversial whether LAC is suitable for patients with T4 colon cancer, due to the difficulty to achieve complete resection (R0) and frequent conversion to open procedure. In some studies, stage T4 was regarded as a risk factor that may lead to poor oncologic outcomes for LAC in comparison to OC [9–11]. However, some comparative analyses claimed that patients with T4 colon cancer yielded similar outcomes from both LAC and OC [12–14].

Although some studies have compared the short-term outcomes and long-term oncologic results of laparoscopy versus open surgery for patients with T4 colon cancer, consistent conclusions for the application of LAC for T4 colon cancer were difficult to draw from these works. What’s more, most of them enrolled heterogeneous diseases including colon and rectal lesions and results were incomparable due to the unbalanced characteristics from retrospective analysis [9, 15–17].

Propensity Score Matching (PSM) is proposed as an effective method to control the bias from retrospective analysis [18, 19]. In this study, we summarized our 10-year experience in a single-institution and performed a PSM process to select a comparable cohort attempting to address the aforementioned controversy. We investigated the outcomes of laparoscopic surgery for patients with pathological T4 colon cancer by comparing the short- and long-term oncologic results of LAC and OC, and evaluated the safety and efficacy of LAC for T4 colon cancer.

## Methods

### Patients

A retrospective study of a maintained colon cancer databases was performed. This database collected information of patients who were diagnosed as colon cancer and treated at Department of General Surgery of Nanfang Hospital of Southern Medical University [20]. All analyzed patients were staged according to the seventh edition staging system proposed by the American Joint Committee on Cancer (AJCC).

**Fig. 1:**
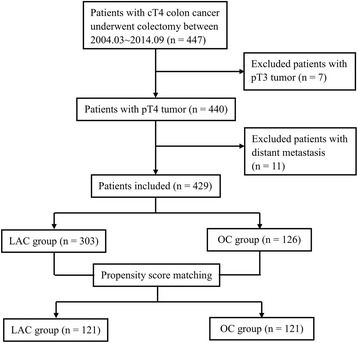
Flow diagram for current study. LAC, laparoscopy-assisted colectomy; OC, open colectomy

Between March 2004 and September 2014, a total of 447 patients with clinical T4 colon cancer who underwent colectomy in our center were collected from the above database [20]. Among 447 patients, 18 patients were excluded, including 7 patients with pathology-proven T3 tumor and 11 patients with distant metastasis. Finally, 429 eligible patients with pT4 colon cancer who underwent either LAC (*n* = 303) or OC (*n* = 126) with curative intent was analyzed (Fig. 1).

### Surgical procedure, adjuvant chemotherapy and follow-up

The decision to proceed laparoscopic or open surgery was made depending on an understanding of the risks and benefits adherent to the laparoscopic and open procedure, without any pressure from the surgeons. All patients underwent a standardized colectomy regimen which included segmental resection of the primary tumor, radical lymphadenectomy and combined resection of invaded adjacent organs if necessary. The bowel resection contained left colectomy, extended left colectomy, transverse colectomy, right colectomy, extended right colectomy, sigmoid colectomy and total colectomy, which was selected adequately depending on primary tumor situation.

All patients analyzed were suggested to receive adjuvant chemotherapy. Personalized adjuvant chemotherapy regimens were prescribed for patients, mainly using capecitabine, oxaliplatin, leucovorin, and irinotecan, according to the updating editions of colon cancer treatment guideline from the National Comprehensive Cancer Network (*NCCN*) guideline [21].

All patients were followed up every 3 months for the first 2 years, 6 months for the next 3 years and annually till the patient’s death. The physical examination, serum tumor marks, chest x-ray, abdominal ultrasonography, gastroendoscopy and (positron emission) computed tomography were chosen adequately for each time of assessments. The last follow-up was in September 2016.

### Measured outcomes

Patients were divided into two groups based on the surgical approach, namely the LAC and OC groups. Conversion to open surgery was defined as an abdominal incision longer than 7 cm or an abdominal incision made different from the previously planned procedure. To minimize the confounding bias, propensity score matching (PSM) was performed [18, 19]. Four covariates (i.e., age, previous abdominal surgery history, comorbidity and primary tumor size) with unbalanced distribution between two groups were used for PSM. The score was estimated using a logistic regression model and greedy matching (ratio = 1:1 without replacement) with a caliper of width 0.2 standard deviations of the logit of the estimated propensity score. Demographic, clinicopathologic, surgical finding and recovery course, and long-term oncologic outcomes were compared between the LAC and OC groups. Long-term outcomes included 5-year overall survival (OS) and 5-year disease free survival (DFS).

### Statistical analysis

Data are presented as mean (±SD) for continuous variables and as frequency (%) for categorical variables. χ^2^ tests (or Fisher’s exact tests, if applicable) were used for comparing categorical data and Student’s *t* tests were applied for continuous variables. Survival curves were estimated by using Kaplan-Meier method and were then compared by log-rank test. A *p* value less than 0.05 (two-tailed) was considered as statistically significant. All statistical analyses were performed with SPSS for Windows, version 20.0 (SPSS, Chicago, IL, USA).

## Results

### Clinicopathologic characteristics of patients

**Fig. 2:**
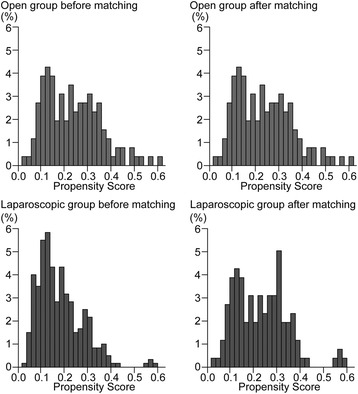
Distribution of propensity score before and after matching

Four hundred and twenty nine patients were finally included in this analysis, including 303 in LAC group and 126 in OC group. The details of patients’ characteristics are presented in Table 1. There were significantly unequal distributions between the two groups in aspects of age, previous abdominal surgery history, comorbidity and primary tumor size. Therefore, PSM was performed by matching these four variables for selection of patients with comparable characteristics. Propensity score distributions of two groups before and after matching are shown in Fig. 2. One hundred and twenty one patients were matched in each group, with no significant difference in clinical and pathological characteristics (Table 1).

**Table 1:** Clinicopathologic characteristics of studied patients before and after matching

	Before matching			After matching		
	LAC (*n* = 303) N (%) or mean (SD)	OC (*n* = 126) N (%) or mean (SD)	*p*	LAC (*n* = 121) N (%) or mean (SD)	OC (n = 121) N (%) or mean (SD)	*p*
Age (year)	55.00 (13.75)	59.46 (14.41)	0.003	56.95 (13.91)	59.61 (14.61)	0.148
Gender (male)	188 (62.0)	80 (63.5)	0.778	76 (62.8)	81 (66.9)	0.501
BMI (Kg/m^2^)	22.46 (3.68)	21.70 (3.53)	0.205	22.04 (2.98)	21.62 (3.54)	0.490
Previous abdominal surgery (+)	35 (11.6)	25 (19.8)	0.024	15 (12.4)	23 (19.0)	0.158
Comorbidity			0.003			0.201
0	227 (74.9)	76 (60.3)		85 (70.2)	74 (61.2)	
1	57 (18.8)	31 (24.6)		26 (21.5)	29 (24.0)	
≥ 2	19 (6.3)	19 (15.1)		10 (8.3)	18 (14.9)	
Neoadjuvant chemotherapy (+)	5 (1.7)	6 (4.8)	0.089	1 (0.8)	6 (5.0)	0.120
Primary tumor size (cm)	5.02 (2.17)	5.50 (2.41)	0.042	5.02 (2.09)	5.49 (2.31)	0.096
Histological differentiation			0.081			0.571
G1~ 2	230 (76.7)	93 (72.1)		94 (77.7)	87 (71.9)	
G3	39 (13.0)	27 (20.9)		20 (16.5)	26 (21.5)	
Gx	31 (10.3)	9 (7.0)		7 (5.8)	8 (6.6)	
Conversion to open surgery (+)	15 (5.0)	–		11 (9.1)	–	
Severe adhesion	5 (33.3)	–		5 (45.5)	–	
Bulky tumor	8 (53.4)	–		4 (36.4)	–	
Technical difficulties	2 (13.3)	–		2 (18.2)	–	
Primary tumor location			0.635			0.235
Caecum	7 (2.3)	5 (4.0)		1 (0.8)	5 (4.1)	
Ascending colon	72 (23.8)	31 (24.6)		32 (26.4)	30 (24.8)	
Transverse colon	78 (25.7)	37 (29.4)		27 (22.3)	35 (28.9)	
Descending colon	34 (11.2)	15 (11.9)		14 (11.6)	16 (13.2)	
Sigmoid colon	112 (37.0)	38 (30.2)		47 (38.8)	35 (28.9)	
Surgical procedure			0.128			0.095
Left colectomy	65 (21.5)	17 (13.5)		28 (23.1)	16 (13.2)	
Extended left colectomy	1 (0.3)	0 (0.0)		1 (0.8)	0 (0.0)	
Transverse colectomy	9 (3.0)	10 (7.9)		2 (1.7)	9 (7.4)	
Right colectomy	112 (37.0)	53 (42.1)		43 (35.5)	52 (43.0)	
Extended right colectomy	18 (5.9)	6 (4.8)		6 (5.0)	6 (5.0)	
Sigmoid colectomy	92 (30.4)	39 (31.0)		39 (32.2)	37 (30.6)	
Total colectomy	6 (2.0)	1 (0.8)		2 (1.7)	1 (0.8)	
Multivisceral resection (+)	152 (50.2)	85 (67.5)	0.100	62 (51.2)	65 (53.7)	0.699
Clinic N stage			0.108			0.095
cN0	169 (55.8)	70 (55.6)		67 (55.4)	66 (54.5)	
cN1	87 (28.7)	45 (35.7)		33 (27.3)	44 (36.4)	
cN2	47 (15.5)	11 (8.7)		21 (17.4)	11 (9.1)	
Pathologic T stage			0.916			0.895
pT4a	194 (64.0)	80 (63.5)		75 (62.0)	74 (61.2)	
pT4b	109 (36.0)	46 (36.5)		46 (38.0)	47 (38.8)	
Pathologic N stage			0.961			0.995
pN0	173 (57.1)	73 (57.9)		69 (57.0)	69 (57.0)	
pN1a	40 (13.2)	18 (14.3)		15 (12.4)	17 (14.0)	
pN1b	43 (14.2)	19 (15.1)		20 (16.5)	19 (15.7)	
pN2a	29 (9.6)	10 (7.9)		11 (9.1)	10 (8.3)	
pN2b	18 (5.9)	6 (4.8)		6 (5.0)	6 (5.0)	
Pathologic TNM stage			0.988			0.977
IIB	124 (40.9)	51 (40.5)		48 (39.7)	47 (38.8)	
IIC	49 (16.2)	22 (17.5)		21 (17.4)	22 (18.2)	
IIIB	45 (14.9)	19 (15.1)		19 (15.7)	17 (14.0)	
IIIC	85 (28.1)	34 (27.0)		33 (27.3)	35 (28.9)

### Surgical findings and short-term outcomes comparison between LAC and OC groups

Before matching, there were 15 (5.0%) patients occurring conversion from laparoscopic to open surgery due to severe adhesion of tumor (5 cases, 33.3%), bulky tumor (8 cases, 53.4%) and technical difficulties (2 cases, 13.3%). And in the matched cohort, among 121 patients in LAC group, 11 converted to open surgery (9.1%) with the following reasons: severe adhesion of tumor in 5 cases (45.5%), bulky tumor in 4 cases (36.4%) and technical difficulties in 2 cases (18.2%).

Surgical findings and short-term outcomes for the propensity-matched cohort are summarized in Table 2. There was no statistically significant difference in mean operating time between the LAC and OC groups, whether for pT4a stage (LAC 148.71 min vs. OC 160.32 min, *p* = 0.153) or for pT4b stage (LAC 165.24 min vs. OC 180.13 min, *p* = 0.273). Although the proportion of patients with intraoperative blood transfusion in two groups was similar for both pT4a (LAC 5.3% vs. OC 8.1%, *p* = 0.533) and pT4b stages (LAC 6.5% vs. OC 8.5%, *p* = 0.716), patients received LAC experienced less blood loss than those received OC (for pT4a stage, LAC 91.80 ml vs. OC 135.68 ml, *p* = 0.002; for pT4b stage, LAC 89.24 ml vs. OC 168.09 ml, *p* = 0.020). Whether for pT4a or pT4b stage, R0 resection was achieved at similar rates between two groups without statistical difference (both *p* > 0.05). For postoperative recovery, the time to first flatus and first liquid intake in the LAC group were significantly shorter than that in the OC group (time to first flatus, LAC 3.35 days vs. OC 4.45 days, *p* < 0.001; time to first liquid intake, 4.28 days vs. 5.62 days, *p* < 0.001) for pT4b stage. However, no significant difference was examined between two groups for pT4a stage, in aspects of time to first flatus (LAC 3.53 days vs. OC 3.94 days, *p* = 0.074) and time to first liquid intake (LAC 4.08 days vs. OC 4.59 days, *p* = 0.070). Shorter hospital stay was observed in LAC group for both pT4a and pT4b stages. (both *p* < 0.05).

**Table 2:** Surgical findings and short-term outcomes for patients after matching

	Pathologic T4a stage (*n* = 149)			Pathologic T4b stage (*n* = 93)		
	LAC (*n* = 75) N (%) or mean (SD)	OC (*n* = 74) N (%) or mean (SD)	*p*	LAC (*n* = 46) N (%) or mean (SD)	OC (*n* = 47) N (%) or mean (SD)	*p*
Operating time (min)	148.71 (44.75)	160.32 (53.73)	0.153	165.24 (60.16)	180.13 (68.71)	0.273
Estimated blood loss (ml)	91.80 (90.25)	135.68 (80.50)	0.002	89.24 (75.87)	168.09 (211.99)	0.020
Intraoperative blood transfusion (+)	4 (5.3)	6 (8.1)	0.533	3 (6.5)	4 (8.5)	0.716
Residual tumor			0.094			0.082
R0	72 (96.0)	67 (90.5)		42 (91.3)	36 (76.6)	
R1	3 (4.0)	2 (2.7)		1 (2.2)	7 (14.9)	
R2	0 (0.0)	5 (6.8)		3 (6.5)	4 (8.5)	
Time to first flatus (day)	3.53 (1.29)	3.94 (1.42)	0.074	3.35 (1.12)	4.45 (1.64)	< 0.001
Time to first liquid intake (day)	4.08 (1.35)	4.59 (1.97)	0.070	4.28 (1.44)	5.62 (1.80)	< 0.001
Postoperative hospital stay (day)	9.04 (3.90)	12.51 (5.28)	< 0.001	10.80 (6.82)	13.94 (7.93)	0.044

### Long-term outcomes

**Fig. 3:**
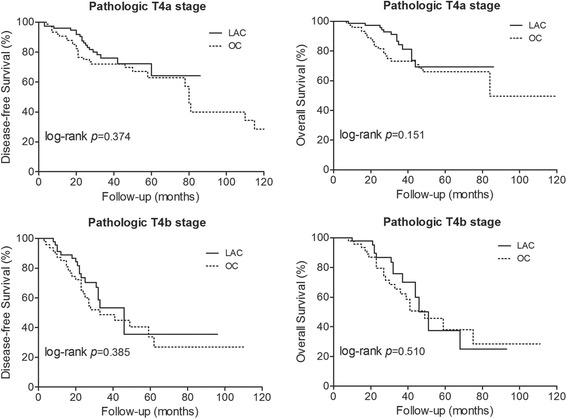
Kaplan-Meier curves comparisons for disease free survival and overall survival between LAC and OC groups according to pathologic T stage

Two hundred and forty two patients were followed up with a mean duration of 43.7 months (range, 7-136 months). OS and DFS curves for pT4a and pT4b stages are shown in Fig. 3. For DFS and OS, no significant difference was examined between LAC and OC groups, whether for pT4a or pT4b stage. The 5-year DFS rate was 64.2% for pT4a stage and 35.5% for pT4b stage in LAC group, and 62.9% and 33.7% in OC group for pT4a (*p* = 0.374) and pT4b (*p* = 0.385) stage respectively. For 5-year OS rates, two groups were also similar in pT4a stage (LAC 69.2% vs. OC 66.0%, *p* = 0.151) and pT4b stage (LAC 37.5% vs. OC 38.1%, *p* = 0.510).

## Discussion

Recently, a meta-analysis published 5 studies on comparing the oncologic outcomes following laparoscopic versus open resection of pT4 colon cancer [22]. It concluded that LAC appears to be safe for selective patients with pT4 tumor, which is similar to ours. This analysis tried to adjust for confounders by using matching method. In its one concluded study in which matching was performed, it seems that several covariates, i.e. age, sex, tumor stage, body mass index and ASA score were controlled, however, some more critically factors, such as tumor size, which is an important factor causing intraoperative conversion for T4 tumor, were failed to controlled at baseline. In this study, PSM method was used to control the bias and inherent limitations in a retrospective study, which is considered to be a robust design among non-randomized studies [23]. Similar baselines in two groups were examined in our matched cohorts, indicating the efficacy of a PSM process. With the faster recovery and no observed-adverse oncologic outcomes, this study suggests that laparoscopy appears to be a safe and efficacious approach for patients with pT4 colon cancer in centres with experienced expertise in minimally invasive surgery.

At present, application of laparoscopic colectomy is more and more popular, but for patients with T4 colon cancer. There is lack of randomize clinical trials to assess the safety and efficacy and it still remains a controversy. One of mainly reasons is the higher proportion of patients with T4 disease experiencing conversion to open surgery from laparoscopy. Usually, conversion to open surgery is performed if there is any doubt to ensure the safety of a laparoscopic approach or the technical accommodation. According to the results from an earlier randomized controlled trial (COLOR) in 2009, up to 50% of patients were converted to open surgery and it was recognized that T4 lesions increased the risk of conversion to open surgery [10]. However, according to results reported recently, the conversion rate from laparoscopy to open surgery in T4 colorectal cancer has reduced to an acceptable rate ranged from 7.1 to 24.7% [12, 13, 24–26]. The common reasons for conversion include tumor fixation, severe bleeding, inadequate visualization, large tumor and invasion of adjacent organ. In our matched cohort, 11 patients underwent laparoscopic procedures were converted to open surgery (9.1%). With the development of laparoscopic techniques and equipment, the trend towards lower conversion rate during LAC procedure could be explained by the accumulated experience of laparoscopic surgery [24].

Consequences induced by conversion have been discussed, including extended incision, higher morbidity, longer operating time and perhaps poorer oncologic outcomes [3, 27–29]. It was considered that extended surgery and altering the surgeon’s routine practice in cases of locally advanced malignancy probably increased morbidity [30, 31]. Conversion is believed to prolong operating time and increase blood loss because the approach experiences more surgical procedures. Although conversion impairing the long-term prognosis was reported in several analyses, most previous studies did not show statistically significant worse oncologic results in the converted group [14, 23]. In this study, outcomes for converted procedure were failed to evaluate since a small sample size in such group. Certainly, the impact of conversion on postoperative outcomes should be evaluated in a large-scare study, and whether converted surgery would jeopardize long-term prognosis still remains controversial.

On the premise of an acceptable converted rate in our study, the short-term courses showed a faster recovery of the LAC group compared with the OC group. Difference of operating time was not found between the LAC and OC groups in our study as well as in most previous studies, reflecting the comparable feasibility of the surgical procedures with the accumulated experience of laparoscopy [14, 23, 24, 32]. Patients in the LAC group lost less blood than in the OC group, which is probably because those patients experienced less invasive procedures during the laparoscopic surgery. For patients received LAC, some studies reported more time to first soft diet [15, 16], while our study as well as others observed similar even shorter time to first liquid intake and to first flatus [12–14]. The results could be translated into shorter lengths of postoperative hospital stay, which is similar to the outcomes published previously [12–14, 24, 32]. Overall, similar to other resectable colon cancer, patients with T4 disease received laparoscopic surgery can recover faster than those received open procedure and it suggests that laparoscopy is an alternative for patients with T4 colon cancer.

Another reason for that laparoscopy is concerned to recommend for patients with T4 colon cancer is the possible worse oncologic outcomes due to incomplete resection. R0 resection is considered to be one of most important prognostic factors [33] and the essential determinant for good oncologic outcomes in patients with the surgical management of locally advanced colon cancer [21]. Laparoscopic resection of T4 tumor, especially for T4b stage, was thought to be a demanding procedure of spatial resolution, dexterity and technical skill to accomplish R0 resection. Recent studies showed an acceptable rate of R0 resection ranged from 73.8 to 100% for T4 colon cancer [13, 14, 24–26, 32]. In our study, curative R0 resection could be achieved in more than 90% of cases in the LAC group.

With the suitable R0 resection in this cohort, the long-term oncologic outcomes, including 5-year DFS and OS rates are comparable between the LAC and OC groups. This is consistent with those conclusions demonstrated previously [12–15, 24, 32]. The 5-year DFS rate (64.2% and 35.5% in pT4a and pT4b stages, respectively) and OS rate (69.2% and 37.5% in pT4a and pT4b stages, respectively) for patients received LAC in our study are similar to results reported in previous studies [13, 24]. The outcomes indicate that LAC would not result in adverse long-term oncologic outcomes for patients with T4 colon cancer.

## Conclusions

In this study, our analysis of a propensity-matched cohort concluded that patients with pT4 colon cancer underwent LAC gained some intraoperative advantages, such as less blood loss, and yield faster recovery and comparable oncologic outcomes, including 5-year DFS and OS, when compared with those underwent OC. The rate of conversion to OC, which is the main concern about the application of LAC, was acceptable in this cohort. These findings suggest that LAC appears to be safe for selected T4 colon cancer patients in centres with expertise in minimally invasive surgery.

## Abbreviations

DFS: disease free survival
LAC: laparoscopy-assisted colectomy
OC: open colectomy
OS: overall survival
PSM: Propensity Score Matching
RCTs: randomized controlled trials
